# Cholesterol Alters the Dynamics of Release in Protein Independent Cell Models for Exocytosis

**DOI:** 10.1038/srep33702

**Published:** 2016-09-21

**Authors:** Neda Najafinobar, Lisa J. Mellander, Michael E. Kurczy, Johan Dunevall, Tina B. Angerer, John S. Fletcher, Ann-Sofie Cans

**Affiliations:** 1 Department of Chemistry and Chemical Engineering, Chalmers University of Technology, SE-412 96 Gothenburg, Sweden; 2 Department of Chemistry and Molecular Biology, University of Gothenburg, SE-412 96 Gothenburg, Sweden

## Abstract

Neurons communicate via an essential process called exocytosis. Cholesterol, an abundant lipid in both secretory vesicles and cell plasma membrane can affect this process. In this study, amperometric recordings of vesicular dopamine release from two different artificial cell models created from a giant unilamellar liposome and a bleb cell plasma membrane, show that with higher membrane cholesterol the kinetics for vesicular release are decelerated in a concentration dependent manner. This reduction in exocytotic speed was consistent for two observed modes of exocytosis, full and partial release. Partial release events, which only occurred in the bleb cell model due to the higher tension in the system, exhibited amperometric spikes with three distinct shapes. In addition to the classic transient, some spikes displayed a current ramp or plateau following the maximum peak current. These post spike features represent neurotransmitter release from a dilated pore before constriction and show that enhancing membrane rigidity via cholesterol adds resistance to a dilated pore to re-close. This implies that the cholesterol dependent biophysical properties of the membrane directly affect the exocytosis kinetics and that membrane tension along with membrane rigidity can influence the fusion pore dynamics and stabilization which is central to regulation of neurochemical release.

The key regulatory event in neuronal communication is the release of neurotransmitters, which occurs through a process known as exocytosis, the fusion of intracellular transmitter packed vesicles with the plasma membrane. The components of the fusion machinery are in part well characterized, where a major role is played by an assembly of cell and vesicle membrane proteins known as the SNARE-complex. The SNARE-mediated catalysis of lipid mixing and fusion of the two membranes, initiates the exocytosis process and results in the formation of an initial fusion pore that is thought to be a few nanometers in diameter^1^. This narrow pore subsequently either fully dilates, incorporating the vesicle membrane into the plasma membrane resulting in full vesicle content release in what is referred to as “full exocytosis”. Alternatively, a second mode of exocytosis known as “kiss-and-run” occurs, where the fusion pore widens and releases part of the vesicle content before re-closing^2^,^3^. Hence, during the exocytosis process the membrane of the lipidic fusion pore connecting the secretory vesicle membrane and the plasma membrane goes through dynamic high curvature shape transitions. It has been shown that changes in local chemical environment and the physical forces acting upon the cell membrane can alter the mode of exocytotic release^4^,^5^,^6^.

The cell membrane is considered as a two-dimensional fluid in which its constituents of proteins and lipids can diffuse^7^. Hence, for a membrane in a fluid state, its material properties display both flexibility and robustness, and due to membrane elasticity, can display many different shape transitions depending on forces acting upon its surface. Energy models can be used to predict the dynamic membrane shape transitions resulting in minimal energy cost^8^,^9^,^10^. For membranes to deform, the two major energetic barriers are the resistance for membrane bending and stretching. Bending of the membrane creates curvature and is influenced by membrane composition and local environment^11^,^12^, while stretching the membrane results in a high energetic cost by decreasing the lipid density in the membrane. Calculations on membrane energetics during the exocytosis process, demonstrate that fusion pore formation and pore dilation are high energy shape transitions, and lateral membrane tension is thought to be a major force in driving these processes^13^,^14^,^15^. Membrane tension is a good candidate for driving expansion of the fusion pore at the later stages of the exocytosis process as this tension is affected by local membrane curvature and at this stage the membrane is highly curved.

Theoretical modeling demonstrates that relaxation times for tension gradients is very fast, occurring within milliseconds, while relaxation of shape changes is slower and estimated on the order of seconds^16^. Experimentally the effect of membrane tension on fusion pores dynamics has been displayed to induce flickering of the fusion pore before dilation in protein-free systems^17^, dictating the mode of exocytotic release both in a cell model and in live cells^6^,^18^, to act as a mechanical feedback for regulation of exocytosis activity^19^,^20^, and as a stress component for secretory granules to induce exocytosis fusion^21^.

While many of the proteins involved in exocytosis have been identified and characterized, the critical role of membrane lipids has not yet been clearly defined. Cholesterol, an abundant sterol in eukaryotic cell membranes^22^, has been shown to be a critical regulator of exocytosis, likely in part by changing the properties of the cell membrane^23^,^24^,^25^,^26^,^27^,^28^. In contrast to phospholipids, cholesterol translocates rapidly between the two leaflets of the plasma membrane^29^. By its heterocyclic ring structure, cholesterol preferentially partitioning in the center region of the hydrophobic tails groups of the lipid bilayer and enhances the packing density of lipids in the membrane, increasing membrane stiffness and mechanical strength^30^,^31^. Hence, modulation of the biophysical properties of the membrane by cholesterol reduces membrane fluidity and adds resistance to membrane deformation. Contrary to what might be expected though, cholesterol has been shown to facilitate exocytosis. This has been hypothesized to be due to its ability to modulate membrane curvature, where the spontaneous negative curvature of cholesterol is believed to favor negative curvature regions of the membrane such as the cytosolic side of the fusion pore^32^,^33^.

It is however arduous to extract the influence of cholesterol on the membrane dynamics of exocytosis in a living cell because cholesterol is also intimately involved with membrane proteins. Altering the amount of cholesterol in the cell membrane has been shown to rearrange the cytoskeleton, influence actin polymerization^34^, alter the molecular organization of synaptic membranes causing re-localization of secretory proteins^35^,^36^,^37^, and to alter vesicle motion and docking^24^.

Here, we dissect the influence of cholesterol on membrane biophysics through the employment of two previously developed artificial cell models to give context to the resulting effects to the exocytosis process. In both model systems a microinjection pipette filled with a dopamine solution is electroporated through the vesicle membrane and used to pull a lipid nanotube from the opposite membrane to the inside of the vesicle. The tube is then inflated to form a dopamine filled daughter vesicle connected to the outside of the large vesicle through a nanotube. This assembly, consisting of a neurotransmitter filled daughter vesicle, a nanotube, and the initial vesicle simulates the secretory vesicle, the fusion pore, and the plasma membrane structure formed during exocytosis (Fig. 1a). By continuous inflation of the daughter vesicle it grows until reaching the “cell plasma membrane” upon which the nanotube expands releasing the vesicle content and mimicking the final stages of exocytosis.

The giant liposome based cell model is a low membrane tension system that mimics full vesicle release (Fig. 1b)^38^. The second cell model is constructed from PC12 cell plasma membrane blebs and is a high membrane tension system that also in difference to the liposome cell model in addition to a lipid bilayer also contains a large fraction of proteins in the membrane (Fig. 1c)^39^,^40^. This system has previously been shown by us to mimic two alternate modes of exocytosis, full (Fig. 1d,i) and partial vesicle release (Fig. 1d,ii)^6^. The release mode in this cell model is regulated by alterations in membrane tension.

By placing a microelectrode in close proximity to the cell model membrane during exocytosis, the neurotransmitter release events can be recorded as individual amperometric spikes. These spikes provide kinetic information of single release events^41^,^42^ such as the peak rise time (T_rise_) which represents the opening of the fusion pore, the half width (T_½_) defining the duration of the exocytotic event and the current spike fall time (T_fall_) which relates to the diffusion of molecules toward the electrode in addition to sealing of the vesicle opening to reform the initial fusion pore. In this work, amperometric recordings of dopamine release from two different cell models exhibiting high and low membrane tension was employed to evaluate the influence of cholesterol on two distinct modes of exocytosis. The kinetic information from the current spike characteristics was used to deduce that cholesterol influences the fusion pore dynamics by stiffening the membrane of the fusion pore and widening the pore size, which is in agreement with recordings performed at live cells^43^,^44^.

## Results

In this study, we employed two cell models for exocytosis to study the influence of cholesterol on membrane dynamics during the exocytosis process by altering the membrane cholesterol content in a controlled fashion. In experiments employing a liposome based cell model the cholesterol concentration was altered through the addition of different cholesterol ratios to the soy bean lipid extract mixture during liposome preparation, whereas in the bleb cell model, the cholesterol membrane content was either reduced or augmented by using β-cyclodextrin or cholesterol saturated β-cyclodextrin respectively.

### In the liposome cell model cholesterol decelerates vesicle content release in a concentration dependent manner

**Figure 2:**
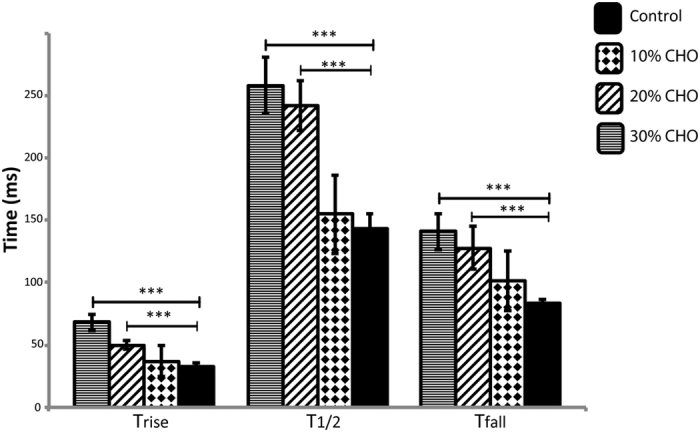
The effect on vesicle release kinetics by adding 10, 20 and 30% cholesterol to the membrane displayed by the current spike parameters T_rise_, T_1/2_, and T_fall_ from amperometric recordings of single vesicle dopamine release from the giant liposome cell model system. Data is collected at single model cells for each cholesterol concentration (n = 10) and is expressed as average ± standard deviation (STD). ***p < 0.001 using a two-tailed unpaired student’s t-test.

The liposome cell model system was employed to investigate the effect of cholesterol on the kinetics of vesicle release and fusion pore expansion demonstrating conditions of low membrane tension. Various amount of cholesterol (10, 20 and 30%) were added to the liposome membrane and vesicle release from the different cell model preparations was recorded. In Fig. 2, the kinetic parameters, T_rise_, T_½_, and T_fall_ of the amperometric peaks are presented for control experiments, where no cholesterol was added to the liposome preparation, and for cell model experiments with augmented cholesterol. The data shows that the kinetics of vesicle release slows down with increasing concentrations of membrane cholesterol, influencing both the fusion pore expansion and membrane resealing phases of the event.

### Modifying membrane cholesterol content alters the kinetics of full and partial release in the bleb plasma membrane cell model

**Figure 3:**
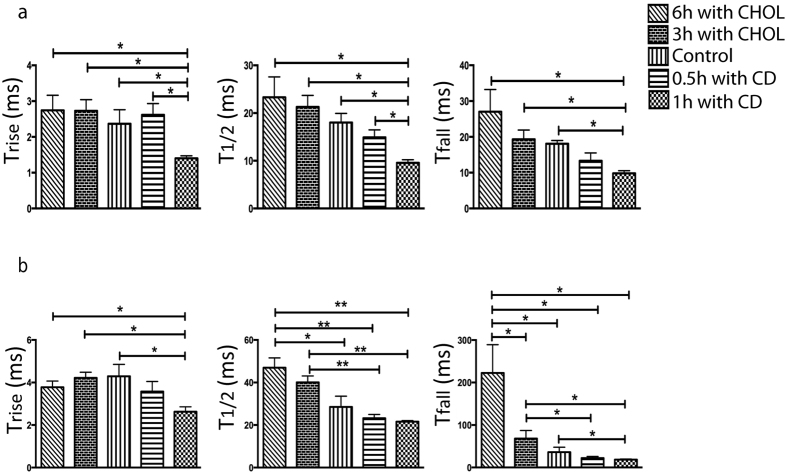
The kinetic current spike parameters T_rise_, T_1/2_, and T_fall_ for (**a**) full and (**b**) partial release recorded at the bleb cell model with or without adding or reducing membrane cholesterol content. Data are collected at single model cells during full release (n = 3) and partial release (n = 6) and is expressed as average ± square error mean (SEM). *p < 0.05 and **p < 0.01, using a two tailed unpaired student’s t-test.

In the plasma membrane bleb cell model the concentration of membrane cholesterol was altered by incubation of the PC12 cells with β-cyclodextrin for 30 or 60 minutes, or saturated cholesterol β-cyclodextrin for 3 or 6 hours. During amperometric recording, the mode of release in the bleb cell model, whether full or partial, was simultaneously distinguished visually using DIC microscope video recordings. In Fig. 3, the kinetic data from amperometric recordings was categorized for full and partial vesicle release and is presented under conditions; where the composition of the cell membrane was not altered, or either enriched or depleted with cholesterol. For full vesicle release, all kinetic parameters are significantly faster when membrane cholesterol is depleted by incubations with β-cyclodextrin (Fig. 3a). In contrast, the addition of cholesterol to the membrane by incubation with cholesterol saturated β-cyclodextrin slowed the release kinetics, however, it was not a significant detected decrease in speed. In recordings of partial release mode (Fig. 3b), the rate of pore dilation (T_rise_) was increased when membrane cholesterol was depleted, and was unaffected by augmenting the membrane cholesterol content. In contrast, a significant effect was observed for the current spike parameters T_½_ and T_fall_, where the rate of the events appears faster by cholesterol depletion and slower with increasing membrane cholesterol content.

### In partial release, fusion pore dynamics are influenced by membrane cholesterol concentration

**Figure 4:**
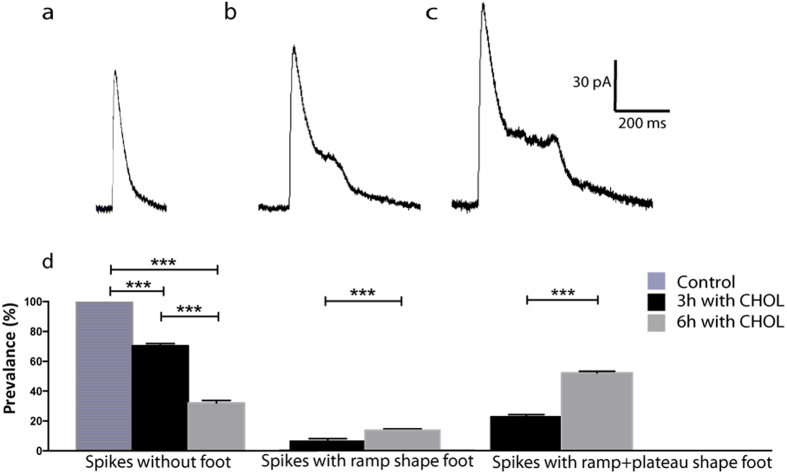
During recordings of partial vesicle release in the cell bleb model, three types of amperometric current spikes were observed after cholesterol incubations: (**a**) spike without post spike foot, (**b**) spike with “ramp” shaped post spike foot, and (**c**) spike with “ramp+plateau” shaped post spike foot. (**d**) The probability of observing different types of spikes is presented as a function of cholesterol incubation time at cells (n = 5). Data are expressed as average ± square error mean (SEM). ***p < 0.001 using a two-tailed unpaired student’s t-test.

Following augmentation of membrane cholesterol at the bleb cell model the membrane dynamics of the fusion pore was highly affected during partial release. This is apparent from the observation of current spikes with three different types of shape characteristics: spikes with no foot (Fig. 4a), spikes with a “ramp” shaped post spike foot (Fig. 4b), and spikes with a “ramp + plateau” shaped post spike foot (Fig. 4c). From our observations the frequency and magnitude of post spike features seem to be directly correlated to the concentration of cholesterol in the membrane. With no additional cholesterol added to the membrane no post spike features were detected, while after 3 or 6 hours of cholesterol incubation 5.8 ± 1.6 and 13 ± 1 percent of the spikes displayed “ramp” shaped post spike feet and 23.9 ± 1.6 and 54.8 ± 1.2 exhibited “ramp + plateau” shaped post feet respectively (Fig. 4d).

**Figure 5:**
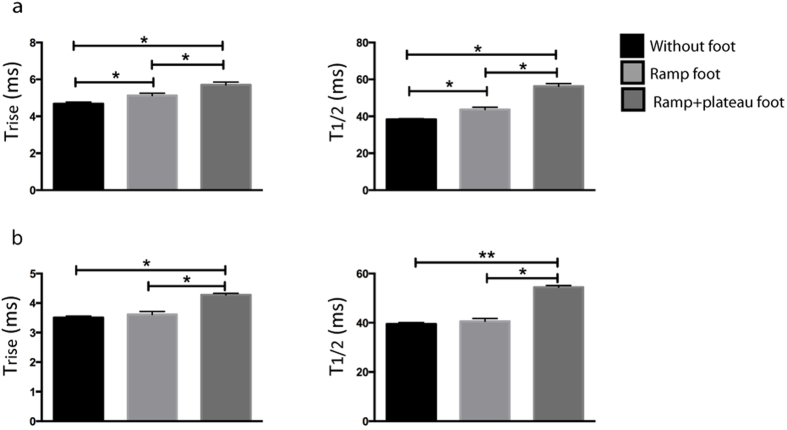
The kinetic parameters T_rise_, and T_1/2_ were categorized for the three different types of spikes detected during recordings of partial vesicle release in the bleb cell model after (**a**) 3 hours and (**b**) 6 hours of cholesterol incubation of cells (n = 5) for each condition. Data is expressed as average ± square error mean (SEM). *p < 0.05 and **p < 0.01 using a two-tailed unpaired student’s t-test.

The enhancement of cholesterol in the membrane tended to hinder pore closure, and increased the probability for the pore to stay open longer and gave rise to a plateau shaped post spike foot. The kinetic parameters T_rise_ and T_1/2_ for the three different types of spikes detected during partial release at the cholesterol augmented bleb cell model were analyzed separately and showed that the delay in pore closure goes hand in hand with slower membrane dynamics of the overall release event (Fig. 5). It is worth noting that a train of events from a single bleb can display all the three different spike shapes depending on the location of micromanipulation of the bleb membrane.

**Figure 6:**
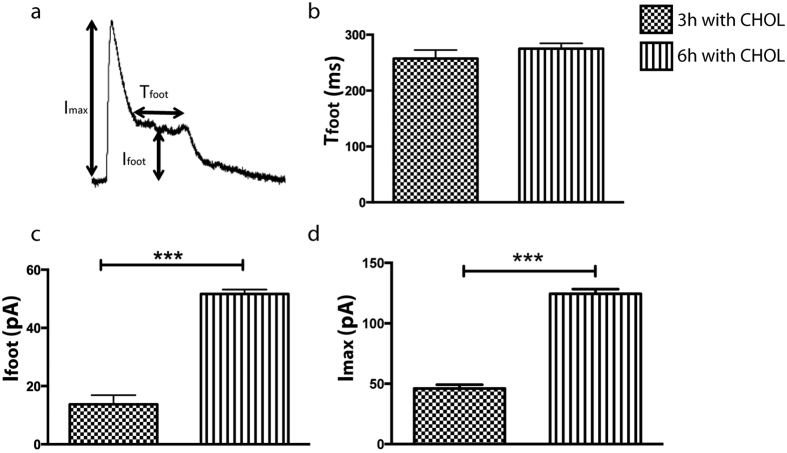
(**a**) A representative current spike with a “ramp + plateau” shaped post spike foot, illustrating the spike parameters analyzed for recordings of partial vesicle release after 3 and 6 h of cholesterol incubations at the bleb cell model: the duration of the post spike foot (T_foot_), the current foot amplitude (I_foot_) and the maximum current spike amplitude (I_max_). Recordings were performed at cells (n = 5) for each condition. Data is expressed as average ± square error mean (SEM). ***p < 0.001 using a two-tailed unpaired student’s t-test.

These extended events imply that an increase in cholesterol concentration influences the stability of the enlarged dilated pore during partial vesicle release and causes a delay in pore closure that allows for a prolonged leakage of neurotransmitters through the stabilized larger pore. This is recorded as the post spike current plateau, which ceases following constriction to a narrow nanotube. To characterize this membrane dynamic effect, we chose to examine two parameters that define post spike plateau foot features. First we considered the duration of the foot (T_foot_), which represent the time the intermediate size pore is stabilized before it collapses back into a narrow lipid nanotube. Secondly, we monitored the amplitude of the foot (I_foot_), which provides relative information on the size of the stabilized pore when compared to the maximal current amplitude (I_max_) that relates to the size of the initial enlargement of the pore when partial vesicle content release is triggered. As shown in Fig. 6, altering the cholesterol content of the membrane affects both the I_max_, and I_foot_ indicating that the size of the pore during enlargement in partial release increases with the membrane cholesterol concentration. In contrast, the membrane cholesterol concentration had no significant effect on the lifetime of the enlarged pore before closing.

## Discussion

In this study we have used two artificial cell models that displays two different modes of exocytosis, full and partial vesicle release, to examine the role of cholesterol on the kinetics of exocytosis with special interest in fusion pore dynamics. The advantage of studying exocytosis using artificial cell models is the reduced complexity of the model cell compared to living cells. Amperometric recording of individual vesicle release of neurotransmitters from these model cells provides kinetic information on fusion pore dynamics at the later stages of exocytosis.

We find that cholesterol slows down the kinetics of neurotransmitter release for both modes of exocytosis. This deceleration can be explained by an increase in membrane rigidity due to augmentation of cholesterol. Studies on secretory cells have also shown that modulating the physical properties of the membrane by alteration of the cellular membrane cholesterol concentration can affect the kinetics of vesicle release in similar manner as displayed in our cell model^24^,^26^,^28^. In addition, through the recording of partial release from the bleb cell model we observed that the shift in membrane cholesterol content influencing the membrane viscosity and rigidity also affected the fusion pore dynamics.

Amperometric recordings of partial release following cholesterol augumentation were found to display features similar to the post spike feet that have been detected in amperometric recordings of dopamine release from PC12 cells^45^. These post spike feet are believed to correspond to leakage of neurotransmitters through a fusion pore that is closing before the vesicle has fully emptied the vesicular content. This suggests that in the model cell during the transition of an enlarged pore structure through which there is a flow of neurotransmitter, the pore goes through an intermediate stage where it is stabilized demonstrating a resistance to shrink back into a nanotube. The increased occurrence of post spike feet by the enhancement of membrane cholesterol concentrations, clearly shows that elevation of membrane cholesterol alters the dynamics of the fusion pore by stabilizing the enlarged pore during the closing process. Hence, enrichment of cholesterol seems to enhance membrane rigidity in the cell model membrane. Studies on cells are in agreement with our findings^24^,^26^,^27^ and specifically one study on platelets has shown that cholesterol increases the percentage of total post spike feet in partial release^43^.

In our model, inflation of the daughter vesicle causes stretching of the membrane, resulting in a lateral tension gradient from daughter vesicle to plasma membrane. This stage is very costly in terms of energy, and the membrane tries to overcome this by a flow of lipid from the plasma membrane toward the daughter vesicle. Adding cholesterol decreases the fluidity and enhances bending rigidity to the bleb cell model membrane. This slows down the lipid flow toward the daughter vesicle, adds resistance to membrane stretching and shape transitioning and results in affecting the fusion pore dynamics. For partial release events, which previously were shown to occur when the membrane is maximally distended, there is no extra lipid available to relax the membrane tension. Instead, the stretching of the membrane forces the narrow pore connecting the daughter vesicle and the cell model plasma membrane to expand in to an enlarged pore in order to release the lateral tension. Around a critical length of a narrow membrane tube, membrane tension and membrane bending has been shown to be playing close to an equal role and by competing for size and shape of a membrane neck displaying a bistability behavior^46^. Although, most often membrane energetics force tension relaxation by acting fast, membrane shape adjustments require more time^16^. By enhancing the cell model membrane rigidity membrane relaxation is slowed down, leading to the stalling of the vesicle in an open state, resulting in the recording of a post spike foot, a feature that was never seen in control membranes.

The post spike foot current amplitude is directly related to the size of the pore during release. The foot current amplitude is larger with higher amount of membrane cholesterol, suggesting that a more rigid membrane is more difficult to bend, forcing the pore to stay open at bigger size. Similar results have been observed in live cells, where cholesterol creates larger fusion pores with similar dynamics as seen here^43^,^44^,^47^. The duration of the foot can in turn be related to the time it takes for the pore to shrink down in to a nanotube and it does in contrast not change with higher amounts of membrane cholesterol. It seems therefore that the fluidity of the membrane reaches a threshold already after three hours of incubation time, where the fluidity of the membrane is low enough and/or bending rigidity is high enough to hinder the immediate relaxation in terms of shape transitioning of the membrane following release.

An interesting observation was that, depending on which part of a bleb membrane was manipulated to inflate a dopamine filled vesicle, all three categories of current spikes could be detected within the same bleb. This indicates the existence of regions with different rigidity within a single bleb plasma membrane and we believe that this might be because the added cholesterol has not been homogenously distributed^48^. This raises the important question whether adding cholesterol to the membrane cause phase separation and result in microdomains of co-existing fluid phases. Liposome experiments have shown that increasing the concentration of cholesterol into the membrane can either lower or raise the transition temperature depending on the lipid membrane composition^49^. However, in our experiments, fluorescence imaging showed that phase separation in blebs with or without incubations of cholesterol, was only observed if the temperature was lowered to 8 °C while no phase separation was observed at room temperature. Hence, these experiments that are performed at 37 °C are far from the transition temperature and therefore adding cholesterol does not cause phase separation in these cell model membranes (supplementary data). An alternate explanation to the observation can be the high membrane tension, since in previous work using liposomes, it has been shown that applying tension to membranes can induce phase domains^50^. Relating to this theory, in our earlier work we have shown in the bleb cell model that an increase in membrane tension above a certain threshold switches the mode of release from full to partial^6^. Hence, this added tension, which switches the exocytosis mode, might also affect membrane transition temperatures and may induce cholesterol membrane concentrations to become heterogeneous. These potential local cholesterol variances might contribute to the result of differences in kinetics and shape characteristics of the current spikes between blebs and within the same bleb. However more investigation is required to confirm this hypothesis.

Although previous studies have focused on the role of cholesterol in membrane protein solubility and localization, we show here, that the isolated cholesterol dependent biophysical properties of the membrane are important, where the concentration of membrane cholesterol is directly affecting the exocytosis kinetics and that membrane tension modifies the influence of cholesterol on fusion pore dynamics. These experiments show that the concentration of cholesterol affects the membrane dynamics in terms of exocytosis speed, and that increasing cholesterol concentrations adds resistance to fusion pore dilation and closing. Hence, the concentration of cholesterol in the membrane might also directly affect the rate of the endocytosis process. We can then speculate that perhaps in areas such as the active zone of the plasma membrane in the nerve terminal, where secretory vesicles dock and fuse, membrane cholesterol concentration might be of great importance and therefore needs to be controlled for efficient vesicle fusion and subsequent vesicle membrane recapture. Perhaps biophysical local membrane stiffness at the active zone is also what directs the location for endocytosis, and where ultra-fast endocytosis has been shown to occur at the membrane edges of the active zone, where membrane fluidity is higher due to lower levels of membrane cholesterol and therefore making it easier for membrane invaginations to occur and for proteins to assist in efficiently recapturing the membrane for vesicle recycling.

## Conclusion

In this study two different artificial cell models have been used to investigate the effect of cholesterol on the kinetics and dynamics of the membrane during the exocytosis process. We are able to clearly demonstrate that the concentration of cholesterol in the cell membrane affects the kinetics of release. Furthermore, we show that membrane tension together with the amount of cholesterol has great effects on the fusion pore dynamics. First, in a protein-free and low-tension system that can mimic the exocytosis mode of full vesicle release, we show that membrane cholesterol adds rigidity to the membrane and decelerates the kinetics of vesicle release in a concentration dependent manner. Second, using an artificial cell model constructed from PC12 cell plasma membrane, where two modes of release can be mimicked, we show that increased rigidity in combination with high tension not only influences the kinetics of release, but also the dynamics of the fusion pore is altered. In recordings of partial vesicle release from this cell model, we observe that a higher cholesterol membrane content increases the chance to stabilize the fusion pore and also affects the size of the pore as it dilates and re-closes. Although SNARE proteins and negatively curved lipids might have an effect on the exocytosis process, we here show that the rigidity of the membrane combined with membrane tension can influence the fusion pore dynamics and stabilization. These results show that the cholesterol content directly affects the kinetics of exocytosis through its effect on the biophysical properties of the cell membrane.

## Methods

### Chemicals

Lipids were obtained from Avanti Polar Lipids. All other chemicals were purchased from Sigma-Aldrich (Sweden) and used as received unless it mentioned otherwise.

### Cell culture and formation of plasma membrane blebs

PC12 cells were provided from the American Type Tissue Culture Collection and maintained as previously described^51^. Briefly, cells were cultured on collagen-coated flasks (BD BioCoat™). After every 7 days cells were sub cultured. Cells were grown is RPMI-1640 cell medium supplemented with 10% donor horse serum and 5% fetal bovine serum and the medium was changed every 2–3 days. For experiments, cells were plated on glass bottom dishes (Willco Wells) coated with collagen type IV and cultured for 2–3 days before use. Blebbing solution (25 mM formaldehyde, 2 mM dithiothreitol (DTT), 10 mM HEPES, 2 mM CaCl2 and 150 mM NaCl, pH 7.4) was used in order to induce the formation of plasma membrane blebs for 30 minutes. After incubation, the cells were washed three times in HEPES buffer (10 mM HEPES, 140 mM NaCl, 1 mM CaCl2, 5 mM KCl and 1 mM MgCl2, 10 mM D-glucose, pH 7.4) before further use.

### Depleting and increasing the cholesterol content of cell membrane

In order to deplete the cholesterol from the cell plasma membrane, 10 mM solution of MPCD was dissolved into the cell culture medium to incubate cells for 30 or 60 minutes before blebbing the cells.

The following procedure was used to prepare 10 mL of a 2.5 mM MPCD solution with a 10:l molar ratio of MPCD:cholesterol^52^. A volume of 16 μL of 50 mg/mL cholesterol stock solution dissolved, in chloroform: methanol 1:1 (v:v), was added to a glass tube. The solvent was evaporated under a gentle stream of nitrogen. A mass of 33.45 mg of MPCD (average mol wt 1338) dissolved in 10 mL of cell medium, was then added. The tube was vortexed to bring the dried cholesterol off the wall of the tube and then ultrasonicated for 3 min. This 100% saturated cyclodextrin: cholesterol solution was incubated in a water bath at 37 °C overnight. Immediately before use, the solution was filtered through a 0.45 μm syringe filter (Millipore, Bedford, MA) to remove excess undissolved cholesterol crystals. This solution was used to incubate cells for 3 or 6 hours before blebbing^52^.

### GUV preparation and incubating with cholesterol

Surface-immobilized giant unilamellar liposomes (SBL) were prepared from soybean polar lipid extract as described elsewhere^38^,^53^. Briefly, the lipid extract was first dissolved in chloroform and then dried using a rotation evaporator (Büchi, Switzerland). In the following step buffer solution (5 mM Trizma base/30 mM K3PO4/30 mM KH2PO4/1 mM MgSO4/0.5 mM EDTA adjusted to pH 7.4 with H2SO4) was used to rehydrate the dried lipid film at 4 °C for 24 h and then ultrasonicated for 1 minute. In the day of the experiment, 3 μL of the lipid suspension was transferred onto a glass coverslip (Menzel-Gläser, Braunschweig, Germany; 24 mm × 60 mm, no. 1) and dehydrated in a vacuum desiccator. The slide was then transferred to the microscope for experiments and was rehydrated again with buffer. This method produces giant unilamellar liposomes attached to a multilamellar liposome that can function as a lipid reservoir during these experiments. Augmentations of the SBL preparation were made with 5, 10, 20 and 30 weight % cholesterol.

### Artificial exocytosis in liposome and plasma membrane cell models

All experiments were performed as previously described^6^. Briefly, A glass micropipette (o.d. 1 mm, i.d. 0.78 mm, with filament; Harvard Apparatus, Holliston, MA) was pulled on a commercial filament puller (P-1000; Sutter Instrument Co., Novato, CA) using a Type C program yielding pipettes 0.8–0.5 μm and 40–80 M ohm. In both models, the pipette was electro-inserted into the membrane of vesicle followed by electroporation of pipette through the second membrane of the vesicle. The pipette was then retracted back to the inside of the vesicle, pulling in a plasma membrane nanotube. Hence, this tube connects the inside of the pipette with the outside of the vesicle. By applying a constant backpressure of 40–50 hPa through the pipette, using a FemtoJet microinjector (Eppendorf/Brinkmann Instruments, Hauppauge, NY), the tube was inflated to form a daughter vesicle at the tip of the pipette. Prior to experiments, the pipette was backfilled with 50 mM dopamine dissolved in a buffer solution (5 mM Trizma base, 15 mM K3PO4, 30 mM KH2PO4, 10 mM K2HPO4, 0.5 mM EDTA and 1 mM MgSO4 at pH 7.4) for bleb model and 2 mM dopamine in a buffer solution (5 mM Trizma base/30 mM K3PO4/30 mM KH2PO4/1 mM MgSO4/0.5 mM EDTA adjusted to pH 7.4 with H2SO4) for pure liposome model. When the daughter vesicle reaches the membrane of the artificial cell and fuses into the membrane, the dopamine content of the vesicle is detected by placing an electrode close to the membrane. By applying 700 mV potential the dopamine molecules are oxidizing to dopamine ortho-quinone and an amperometric spike is produced. All the experiments were performed at 37 °C for bleb cell model and at room temperature for the liposome cell model.

### Carbon fiber amperometry

Carbon fiber electrodes were prepared as previously described^6^. Release from both cell models was measured using amperometry at a carbon fiber microelectrode positioned at the exit of the nanotube. The microelectrodes were constructed by aspirating a 5-μm carbon fiber for bleb model and 30-μm carbon fiber for GUV model into a glass capillary (o.d. 1.2 mm i.d. 0.69 mm, no filament; Sutter Instrument Co., Novato, CA). A commercial micropipette puller (PE-21, Narishige, Japan) was used to pull the capillary and producing two fiber-containing pipettes. In the following step a scalpel was used to cut the protruding carbon fiber close to the glass tip and then dipped into freshly made epoxy (EpoTek 301, Billerica, MA) for 3 min. The epoxy was allowed to cure and the electrodes were then polished at a 45° angle on a commercial micropipette beveller (Narishige, Japan) and backfilled with 3 M KCl. All electrodes were tested prior to experiments in 100 mM dopamine solution and only electrodes with stable I–E curves were used during experiments. For amperometric measurements, the electrode was kept at 700 mV vs. a silver/silver chloride reference electrode (World Precision Instruments, Inc., Sarasota, FL) using a commercial instrument (Axopatch 200B; Axon Instruments, Foster City, CA). The signal was digitized at 5 kHz and filtered with an internal low pass Bessel filter at 2 kHz. The signal was displayed in real time (AxoScope 8.1; Axon Instruments) and stored digitally.

### Microscopy

An Olympus IX-71 or IX-81 microscope (Olympus, Melville, NY) with a 40x oil objective (Olympus, UApo/340 40x oil iris, NA 1.35) was used to monitor the experiments. Differential interference contrast (DIC) was utilized for a pseudo-three dimensional appearance and contrast enhancement of the vesicle. An Olympus SC20 digital color camera interfaced to a personal computer with the Cell-A software (Olympus, Hamburg, Germany) was used for visual video recording of the experiments.

## Additional Information

**How to cite this article**: Najafinobar, N. *et al*. Cholesterol Alters the Dynamics of Release in Protein Independent Cell Models for Exocytosis. *Sci. Rep.* **6**, 33702; doi: 10.1038/srep33702 (2016).

## Supplementary Material

Supplementary Information

## Figures and Tables

**Figure 1:**
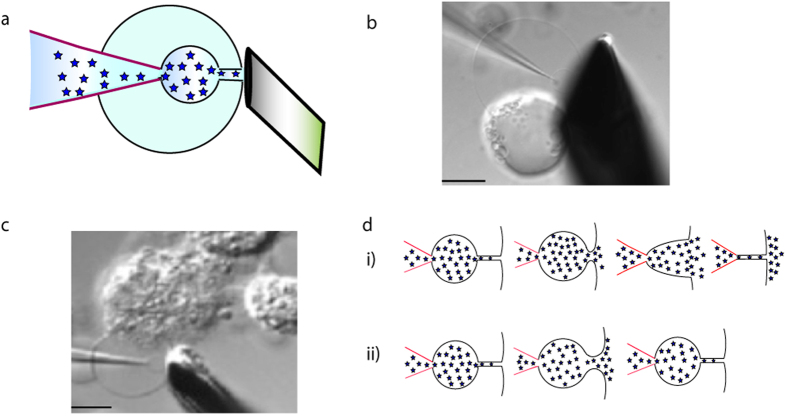
Illustrations of the artificial cell models used in these experiments (**a**) a schematic of the liposome cell model (**b**) a DIC-image of the giant uilamellar liposome cell model, formed from a multilamellar liposome (**c**) a DIC-image of the bleb plasma membrane cell model system and (**d**) schematics representing the two modes of release observed in the bleb cell model: full (*i*) and partial (*ii*). Scale bar is 10 μm.
