# Supplementary material for: Cholesterol Alters the Dynamics of Release in Protein Independent Cell Models for Exocytosis
Source: Sci Rep. 2016 Sep 21;6:33702. doi: 10.1038/srep33702 (PMC5030643; doi:10.1038/srep33702)
Supplement: Supplementary Information [file srep33702-s1.pdf]

Cholesterol Alters the Dynamics of Release in Protein Independent Cell Models for Exocytosis

[†]Neda Najafinobar, [††]Lisa J. Mellander, [†]Michael E. Kurzcy, [†]Johan Dunevall, [††]Tina B. Angerer, [†]John S. Fletcher and [†]Ann-Sofie Cans*

[†]Department of Chemistry and Chemical Engineering, Chalmers University of Technology, SE-412 96 Gothenburg, Sweden

[††]Department of Chemistry and Molecular Biology, University of Gothenburg, SE-412 96 Gothenburg, Sweden

* Corresponding author at: Chalmers University of Technology, Department of Chemical and Biological Engineering, 41296 Gothenburg, Sweden.

E-mail address: cans@chalmers.se

Cell preparation for ToF-SIMS

PC12 cells grown on indium tin oxide (ITO) covered glass slides were incubated with cholesterol MPCD solution for 6 h and thereafter were briefly washed 3 times with a 10 mM ammonium formate solution to remove cell culture media and salts from the surface of the cells. After a 3rd washing step, excess rinsing solution was carefully removed by placing a tissue in contact to the side of the tilted ITO slide until the cells growing at the edges of the glass slide were dry. The majority of the cells in the middle of the slide remained hydrated and intact. Control samples of cells were prepared using the same protocol and eliminating the 6 h incubation step with cholesterol MPCD solution. Cell samples were frozen in liquid nitrogen inside a glove box filled with nitrogen gas and mounted on top of the sample

insertion stage of the ToF-SIMS instrument. Analyzing frozen hydrated cells has the advantage that the cell physiology stays intact and eliminates the need for a chemical fixing step, which may influence the cell chemistry. Freezing cells also prevents intracellular salts from leaking out to the cell surface, which can strongly interfere with the imaging of cholesterol. SIMS is a very surface sensitive technique and use of the glove box minimizes condensation of water on to the surface of the frozen cells that could mask signal from the cell membrane.

ToF-SIMS analysis

ToF-SIMS imaging was performed using a J105 – *3D Chemical Imager* (Ionoptika Ltd, U.K.) and has been described in detail elsewhere[1]. Prior to the analysis the instrument was cooled down and maintained a temperature below 100 K during the entire imaging process. A selected area of cultured cells that were found in a frozen hydrated state and that were not covered in ice at the surface was chosen for the imaging analysis. SIMS-images from the cholesterol MPCD incubated cells and untreated cells were generated with 128 × 128 pixels covering an area of 16384 μm^2 (1 μm^2 per pixel), 40 keV C_{60}^+ was used as the primary ion beam with a primary ion dose of 9.36×10^{13} ions/cm^2 in positive ion mode, mass range 30-950 Da.

Relative quantification shows an increase in cholesterol content of the cell membrane after incubation with saturated cholesterol methyl-beta-cyclodextrin. The increase in amount of cholesterol in the plasma membrane by incubations with saturated cholesterol methyl-beta-cyclodextrin was confirmed by imaging the cell plasma membrane using Time-of-Flight Secondary Ion Mass Spectrometry (ToF-SIMS.) Imaging ToF-SIMS was performed by scanning a primary ion beam over a surface and generating secondary ion mass spectra for

each pixel. This chemical imaging technique is well suited to analyze cholesterol since it produces 2 characteristic fragments, m/z 369.35 ($[M+H-H_2O]^+$, $C_{27}H_{45}$) and m/z 385.35 ($[M+H]^+$, $C_{27}H_{46}OH$) where m/z 369.35 can be observed in higher intensities. Signal from the m/z 369.35 ion is observed to be localized to the cells in the ToF-SIMS image. Summing signal from pixels only where cells are present allows mass spectra to be extracted where the relative intensities of different peaks can be compared between samples. When the PC12 cells were analyzed with ToF-SIMS, the images show cells to be surrounded by and to contain water ice. This confirmed their frozen hydrated state and provided confidence in the sample integrity in the vacuum environment of the mass spectrometer. From the ToF-SIMS images it is apparent that the cholesterol signal at m/z 369.35 (green) is localized to the cells plasma membrane. However, a variation/increase for the treated cells is not directly obvious (Figure S1 a,b). Spectra are generated by summing up the signal from a 2000 μm^2 cell area. Cells were identified in the ToF-SIMS image by the presence of the phosphatidylcholine (PC) headgroup signal of m/z 184.07 ($C_5H_{15}NPO_4$, red in Figure S1 a,b). The PC- headgroup generates a strong peak, since it is the most common constituent of cell membranes. During signal summing, pixels high in ITO substrate signal and/or ice were avoided (m/z 73.05 $(H_2O)_4H^+$, white in Figure S1 and m/z 114.90 In^+, blue in Figure S1 respectively). To avoid variation in the spectra caused by sublet ice and salt coverage variations, that could affect the overall intensity of the signal, the spectra were normalized to the m/z 184.07 signal, that was used as an internal standard. To compare the cholesterol signal we calculated the ratios of the total intensity of a peak window of 0.1 Da. e.g. m/z 369.35±0.05, and divided the signal from the treated PC12 cells (6h Incubation) by the signal from untreated cells (Control). The treated cells showed an average increase in

cholesterol signal of 2.5× for the m/z 369.35 peak and 1.7× for the m/z 385.35 peak. The m/z 369.35 value is considered to be the more reliable value as the signal to noise ratio for the 385.35 signal was very low (Figure S1 c).

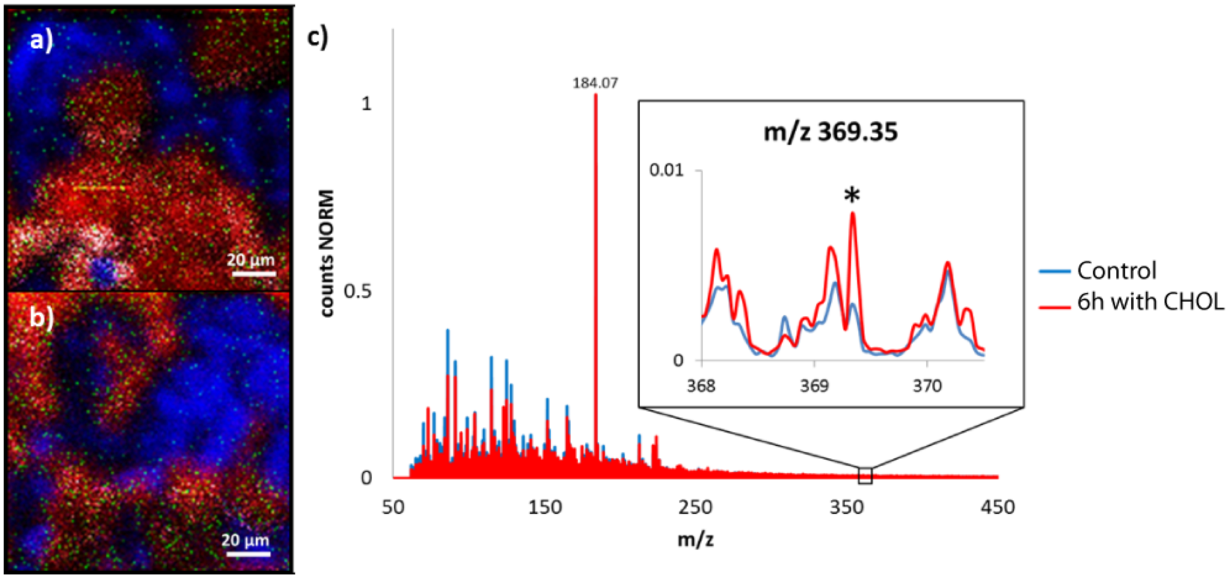

Figure S1. ToF-SIMS overlay images of frozen hydrated PC12 cells on ITO glass a) grown under standard conditions, b) after 6h of cholesterol incubation. Displayed are m/z 114.90 In^+ blue (ITO), m/z 73.05 $(H_2O)_4H^+$ white (ice), m/z 184.07 $C_5H_{15}NPO_4^+$ red (PC-head group) and m/z 396.35 $C_{27}H_{45}^+$ green (cholesterol). C) ToF-SIMS spectra comparison and zoom in on one cholesterol fragment, of frozen hydrated PC12 cells grown under standard conditions (Control) in blue and after 6h cholesterol incubation (6h Incubation) in red, summed up from 2000 single pixel spectra (1 μm^2/pixel). The star indicates the cholesterol-fragment signal at m/z 369.35 ($[M+H-H_2O]^+$, $C_{27}H_{45}$). Spectra were acquired at 100 K using 40 keV C_{60}^+, primary ion dose 9.36 x 10^{13} ions/cm^2, positive ion mode, and have been normalized to their PC-head group signal (m/z 184.07 = signal intensity 1).

Fluorescence microscopy imaging of membrane phase separation in blebbing cells

To study the cell bleb membrane phase transitioning into co-existing fluid phases at different temperature, cells were fluorescently labeled by adding 2 μg/ml Rhodamine-DOPE (Avanti Polar Lipids, Alabama, US) into the blebbing solution and was added to cells in solution during 30 minute of incubation at room temperature before fluorescence imaging similar to previously described[2]. The imaging of phase transitions were performed at three different temperatures: 37 °C, 23 °C, and 8 °C. These imaging experiments were performed on blebbed cells that were incubated with or without cholesterol for either 3 or 6 hours prior to the Rhodamine-DOPE labeling.

The fluorescently labeled bleb membranes were visualized with an IX71S1F-2 Olympus microscope with 60x objective and 620 nm (maximum wavelength) filter set with a 150 W xenon light source. An Olympus SC20 digital color camera interfaced to a personal computer with the Cell-A software (Olympus, Hamburg, Germany) was used to take fluorescent images of the blebs.

For each condition 100 images were taken. At 37 or 21°C, no visible signs of phase separation was observed at cell blebs incubated with cholesterol or at blebs where no cholesterol augmentation was performed. However, lipid fluorophore in blebs demonstrate favored partitioning into the L_d versus L_o phase when cooling the temperature to 8 °C. Phase separation appeared in 3% of the blebs with no cholesterol incubation and in blebs incubated with cholesterol for 3 or 6 hours prior to imaging, 8 % and 10 % of the demonstrated phase separation in the membrane respectively. These results clearly show that phase separation does not occur in cell plasma membrane blebs of cells used in these

experiments that were augmented with cholesterol and at the temperature (37°C) where all our experiments have been performed.

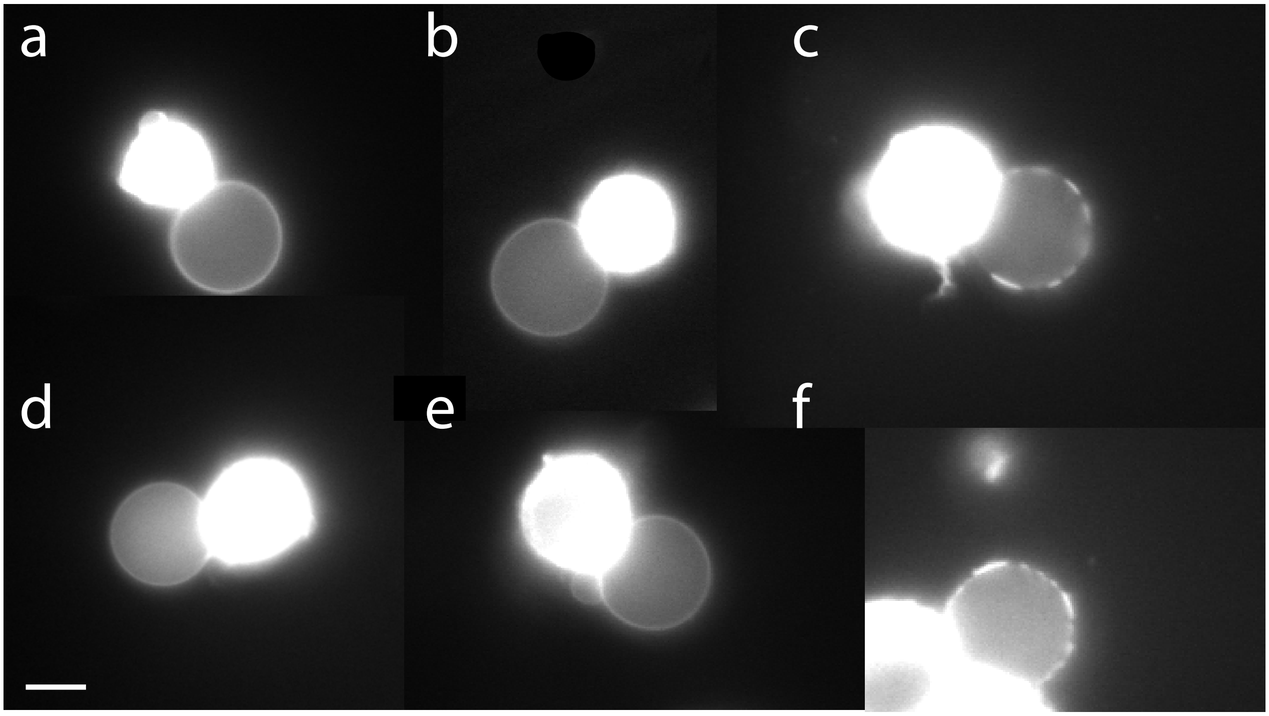

Figure S2. Fluorescence imaging of cell blebs prepared by formaldehyde/DTT treatment and Rhodamine-DOPE labeling to monitor phase separation of the plasma membrane lipids. Cell blebs with no incubation of cholesterol are imaged at a) 37 °C b) 21 °C and c) 8 °C, and after incubation with cholesterol for 6 hours are imaged at d) 37 °C b) 21 °C and c) 8 °C. The scale bar is 20 μm.

References

1. Fletcher, J.S., Rabbani, S., Henderson, A., Blenkinsopp, P., Thompson, S. P., Lockyer, N. P. & Vickerman, J. C. A new dynamic in mass spectral imaging of single biological cells. *Anal Chem.* **80**, 9058-9064 (2008).
2. Baumgart, T., Hammond, A. T., Sengupta, P., Hess, S. T., Holowka, D. A., Baird, B. A. & Webb, W. W. Large-scale fluid/fluid phase separation of proteins and lipids in giant plasma membrane vesicles. *Proc Natl Acad Sci USA.* **104**, 3165-70 (2007).
